# Supplementary material for: A Likelihood Approach for Real-Time Calibration of Stochastic Compartmental Epidemic Models
Source: PLoS Comput Biol. 2017 Jan 17;13(1):e1005257. doi: 10.1371/journal.pcbi.1005257 (PMC5240920; doi:10.1371/journal.pcbi.1005257)
Supplement: S1 File — (TAR.GZ) [file pcbi.1005257.s014.tar.gz › HSPH_Online-SI-Revision/Additions/EnKF08/n100-extreme_noisy/n100-extreme_noisy_EnKF_table8.pdf]

1	R0	{2.76052, 16.3913}	{2.23584, 9.67521}	{{0.781392, 3.46282}, {3., 17.4724}}	{29.2434, 44.2979}	inf	3200.	9223.47	{1000.89, 15 156.8}	226.084	0.419383	newly	1200.81	7340.87	{695., 10 826.}	529.738	0.524107	Reff	0.245824	0.540371	{0.10687, 1.29712}	150.59	ar	92 090.	179 643.	{154 078., 233 290.}	95.0737
2	R0	{2.18202, 15.1734}	{2.74174, 10.482}	{{2.16689, 1.18595}, {3., 16.0785}}	{26.0811, 36.2735}	inf	3327.	6543.98	{1591.54, 9000.78}	129.015	0.270469	newly	1337.	7669.84	{675., 16 473.}	497.837	0.504987	Reff	0.385257	1.04702	{0.524242, 2.16687}	175.15	ar	84 264.	186 108.	{156 828., 249 598.}	120.863
3	R0	{2.57891, 12.5971}	{2.54729, 10.6904}	{{1.88696, 3.26503}, {3., 18.8291}}	{20.1668, 42.549}	inf	1362.	8422.63	{1825.93, 9887.19}	519.744	0.639103	newly	609.	8252.74	{2019., 11 131.}	1255.87	0.846197	Reff	0.262765	0.817704	{0.359433, 1.88369}	213.498	ar	90 302.	176 477.	{149 394., 241 990.}	95.4302
4	R0	{2.5856, 9.28508}	{1.95226, 8.24326}	{{0.860654, 0.905331}, {3., 12.9656}}	{40.8427, 50.7894}	inf	433.	2235.24	{152.403, 2392.08}	442.205	0.607345	newly	254.	2522.09	{68., 2415.}	921.35	0.732462	Reff	0.254526	0.355166	{0.0405745, 0.664445}	92.5563	ar	90 312.	164 193.	{157 198., 162 431.}	81.8067
5	R0	{2.4406, 12.1341}	{2.59095, 14.4609}	{{2.00231, 11.4728}, {3., 17.37}}	{20.8861, 22.8255}	inf	1891.	8694.63	{3077.6, 10 209.7}	367.557	0.592998	newly	894.	4691.64	{1243., 5603.}	433.248	0.595159	Reff	0.297534	0.777617	{0.0959043, 2.00229}	188.401	ar	88 597.	188 484.	{161 610., 259 180.}	112.743
6	R0	{2.72155, 15.4094}	{2.32186, 10.7126}	{{1.27382, 3.45663}, {3., 17.7465}}	{27.3729, 38.3454}	inf	3044.	14 723.5	{2670.73, 41 481.2}	390.717	0.577774	newly	1132.	11 019.5	{1629., 28 116.}	875.342	0.756354	Reff	0.252533	0.462893	{0.0804095, 0.896637}	104.449	ar	91 682.	194 874.	{170 857., 240 980.}	112.554
7	R0	{2.63781, 14.3935}	{2.66225, 8.41221}	{{2.19385, 2.79992}, {3., 11.5222}}	{15.0011, 42.4837}	inf	2374.	4437.67	{284.784, 4132.29}	136.314	0.378118	newly	968.	4527.39	{157., 10 261.}	405.918	0.559723	Reff	0.252043	0.711288	{0.313061, 1.21586}	187.445	ar	91 192.	180 578.	{162 240., 210 609.}	98.0199
8	R0	{2.82391, 16.104}	{2.36579, 5.23024}	{{1.53098, 1.87629}, {3., 7.54451}}	{23.7058, 67.5221}	inf	3239.	1901.81	{296.69, 2272.31}	94.9485	0.673299	newly	1178.	2864.55	{212., 6591.}	213.368	0.440072	Reff	0.232408	0.480811	{0.155578, 0.794279}	117.836	ar	92 664.	157 769.	{149 452., 178 891.}	70.2594
9	R0	{2.80645, 14.6457}	{2.3655, 3.9535}	{{1.37961, 2.67785}, {3., 8.44629}}	{24.6778, 73.0057}	inf	2875.	12 455.8	{2428.61, 42 924.7}	347.428	0.516166	newly	1160.	15 138.1	{3616., 45 073.}	1215.33	0.946859	Reff	0.229399	0.480515	{0.0510312, 0.755595}	142.623	ar	92 567.	205 399.	{175 215., 305 566.}	121.892
10	R0	{1.86213, 5.4271}	{2.33482, 9.29293}	{{1.49733, 2.80602}, {3., 11.9134}}	{41.3938, 90.7597}	inf	95.	4427.	{358.326, 6709.09}	4561.82	1.31693	newly	92.	3844.04	{199., 10 025.}	4080.22	1.21384	Reff	0.460598	0.801301	{0.196071, 1.49952}	99.9024	ar	75 362.	189 838.	{163 406., 239 366.}	151.901
11	R0	{2.27349, 14.6338}	{2.50638, 5.25744}	{{1.45689, 3.36679}, {3., 6.633}}	{33.2162, 64.0733}	inf	2757.	2355.17	{408.588, 8307.01}	74.6134	0.421963	newly	1137.	3208.42	{459., 15 197.}	202.536	0.316202	Reff	0.351982	0.589312	{0.320085, 1.01906}	76.6762	ar	85 805.	166 999.	{150 239., 202 723.}	94.6265
12	R0	{2.77261, 14.1894}	{2.72898, 6.83117}	{{2.31477, 3.7332}, {3., 13.7237}}	{11.2511, 53.1053}	inf	2393.	16 094.9	{3116.91, 35 088.3}	574.277	0.610078	newly	959.	17 287.8	{3496., 53 129.}	1703.56	1.05696	Reff	0.226855	0.918519	{0.33945, 2.31475}	307.337	ar	92 508.	219 249.	{180 049., 294 401.}	137.005
13	R0	{2.65402, 19.3935}	{2.63182, 3.31515}	{{2.31488, 2.42101}, {3., 3.75232}}	{14.6547, 82.9059}	inf	6091.	6193.85	{534.665, 12 434.9}	104.478	0.513952	newly	2008.	15 604.1	{632., 64 008.}	697.078	0.581243	Reff	0.292659	0.995118	{0.355034, 2.31485}	248.572	ar	91 184.	193 289.	{160 429., 270 379.}	111.977
14	R0	{1.75297, 4.45354}	{2.65454, 9.6474}	{{2.1966, 0.797289}, {3., 13.5005}}	{51.4309, 154.374}	inf	33.	6904.26	{1046.84, 9072.23}	20 822.	2.09809	newly	37.	9355.91	{497., 12 133.}	25 186.2	1.92069	Reff	0.50468	0.699232	{0.244415, 2.19658}	76.208	ar	71 263.	173 374.	{150 840., 242 184.}	143.288
15	R0	{1.99928, 7.1501}	{2.59047, 7.80158}	{{1.97617, 4.71811}, {3., 11.8199}}	{30.4947, 48.3372}	inf	273.	1944.72	{929.493, 6303.59}	621.06	0.721445	newly	197.	2504.22	{426., 6071.}	1179.76	0.783209	Reff	0.411872	0.602741	{0.0478786, 1.88763}	89.647	ar	79 602.	185 300.	{165 835., 248 881.}	132.783
16	R0	{2.67398, 10.3134}	{2.55124, 8.16692}	{{1.9801, 3.60714}, {3., 11.5502}}	{18.2455, 28.41}	inf	624.	4657.76	{1244.24, 5504.14}	647.72	0.713528	newly	342.	5330.02	{1289., 8056.}	1459.47	0.902147	Reff	0.237957	0.762456	{0.284771, 1.98008}	222.199	ar	91 289.	187 267.	{164 847., 248 554.}	105.137
17	R0	{2.44634, 16.3226}	{2.24374, 9.59813}	{{1.19602, 6.47125}, {3., 11.1256}}	{34.5352, 41.1973}	inf	3997.	6492.37	{4109.17, 8891.83}	70.9302	0.131928	newly	1461.	4013.35	{1973., 6038.}	181.627	0.329653	Reff	0.317486	0.439315	{0.0544853, 0.829741}	74.2762	ar	88 710.	165 683.	{152 898., 203 502.}	86.7692
18	R0	{2.02168, 10.1676}	{2.52144, 3.84147}	{{1.63739, 3.0367}, {3., 4.46165}}	{37.7072, 62.2185}	inf	1489.	9209.68	{3585.09, 20 699.1}	525.015	0.554485	newly	847.	10 879.9	{2746., 20 755.}	1187.49	0.810186	Reff	0.420772	0.428169	{0.105686, 0.659215}	50.5752	ar	80 299.	199 017.	{180 389., 221 850.}	147.845
19	R0	{2.89969, 15.009}	{2.19266, 9.82182}	{{0.753861, 6.45342}, {3., 14.1968}}	{28.7829, 34.5605}	inf	2686.	7129.06	{2832.76, 3605.18}	175.313	0.240291	newly	1026.	3940.33	{1500., 3143.}	291.264	0.433254	Reff	0.211938	0.488876	{0.130681, 0.65871}	146.103	ar	93 349.	163 270.	{148 294., 192 043.}	74.9024
20	R0	{2.68933, 7.34928}	{2.6369, 7.06003}	{{2.16861, 5.30917}, {3., 9.41042}}	{14.4258, 27.5723}	inf	136.	4621.36	{224.998, 11 194.2}	3299.46	1.27226	newly	80.	5275.38	{150., 14 434.}	6494.87	1.42883	Reff	0.227975	0.645495	{0.205391, 1.58053}	196.573	ar	91 574.	195 557.	{171 314., 278 741.}	113.551
21	R0	{2.15352, 9.28074}	{2.5403, 12.2754}	{{2.02192, 3.87325}, {3., 17.5378}}	{23.7048, 67.1768}	inf	838.	4645.15	{533.846, 7417.1}	471.551	0.775785	newly	504.	2790.85	{652., 5816.}	468.958	0.705999	Reff	0.358733	0.732097	{0.142233, 2.0219}	129.916	ar	83 845.	172 076.	{152 065., 245 371.}	105.231
22	R0	{2.21953, 9.04387}	{2.03983, 4.20521}	{{0.623732, 0.584523}, {3., 12.1103}}	{47.8346, 70.5522}	inf	694.	5680.55	{1603.74, 11 685.}	723.087	0.704393	newly	414.	6572.75	{984., 14 652.}	1491.14	0.935887	Reff	0.343938	0.341102	{0.10465, 0.600447}	56.2695	ar	84 856.	189 150.	{173 289., 241 034.}	122.907
23	R0	{2.21987, 14.5347}	{2.68804, 3.39708}	{{2.25725, 2.89222}, {3., 3.94034}}	{21.0902, 76.6278}	inf	2885.	6671.01	{1980.42, 14 759.}	143.441	0.322149	newly	1192.	12 008.1	{2839., 21 904.}	907.387	0.856685	Reff	0.366057	0.82743	{0.305465, 2.25723}	139.267	ar	85 067.	207 529.	{174 466., 269 962.}	143.959
24	R0	{2.84268, 17.9659}	{2.58409, 5.809}	{{1.93358, 1.51144}, {3., 15.4865}}	{15.8483, 67.6665}	inf	4541.	1210.33	{293.069, 1029.5}	79.3524	0.762999	newly	1477.	2877.7	{302., 1483.}	185.603	0.421	Reff	0.242139	0.429479	{0.0263821, 0.612314}	128.812	ar	92 836.	156 117.	{144 093., 155 380.}	68.164
25	R0	{2.52706, 10.3679}	{2.54883, 7.68014}	{{1.95077, 5.0142}, {3., 10.1141}}	{20.4736, 26.8993}	inf	746.	3216.23	{518.232, 4597.63}	343.269	0.469885	newly	432.	3052.59	{626., 3690.}	614.896	0.615665	Reff	0.269107	0.766918	{0.388803, 1.5453}	190.837	ar	89 623.	184 979.	{167 699., 230 299.}	106.397
26	R0	{2.62835, 19.1088}	{1.78769, 4.29827}	{{0.233005, 3.49609}, {3., 5.84001}}	{47.8212, 77.5063}	inf	5501.	5001.34	{1063.17, 9663.53}	93.6948	0.451315	newly	1758.	4924.53	{915., 8766.}	197.873	0.3311	Reff	0.292378	0.338975	{0.0370036, 0.535085}	87.6521	ar	90 908.	184 363.	{167 281., 232 413.}	102.801
27	R0	{2.76831, 19.4155}	{2.71892, 9.48523}	{{2.21367, 7.6456}, {3., 11.0999}}	{11.8272, 51.1461}	inf	4502.	8850.64	{2391.52, 9398.57}	115.172	0.248396	newly	1418.	7065.23	{1502., 16 142.}	400.634	0.495592	Reff	0.252857	0.816403	{0.219823, 2.21364}	234.71	ar	92 175.	200 405.	{177 938., 262 895.}	117.418
28	R0	{2.96609, 16.8903}	{2.68894, 10.7132}	{{2.13595, 3.91317}, {3., 14.4005}}	{10.8072, 36.5717}	inf	3132.	7862.04	{1405.41, 6174.99}	175.819	0.377307	newly	1123.	6173.55	{969., 6517.}	462.243	0.491765	Reff	0.198639	0.567426	{0.108102, 0.887069}	201.672	ar	94 024.	179 030.	{157 449., 218 471.}	90.4085
29	R0	{2.17046, 14.39}	{1.89738, 4.50021}	{{0.172308, 2.85904}, {3., 5.7114}}	{59.2096, 68.7269}	inf	3337.	5717.07	{2522.07, 3632.79}	82.7464	0.120795	newly	1397.	6103.77	{2597., 4487.}	337.203	0.412754	Reff	0.390705	0.279073	{0.026356, 0.704667}	72.4905	ar	84 001.	179 882.	{167 266., 219 744.}	114.142
30	R0	{2.88772, 12.4647}	{2.06693, 8.35446}	{{0.877148, 3.67788}, {3., 10.8042}}	{32.7783, 39.1898}	inf	1402.	2904.57	{1863.27, 4897.6}	111.825	0.27925	newly	651.	2113.17	{1090., 4324.}	228.966	0.457868	Reff	0.203498	0.46345	{0.06388, 0.872364}	158.943	ar	93 273.	172 640.	{162 320., 213 815.}	85.091
31	R0	{2.46831, 8.14308}	{2.6437, 4.22474}	{{1.92029, 3.26459}, {3., 6.15168}}	{21.7589, 48.1186}	inf	300.	2245.8	{1683.12, 1683.12}	648.676	0.759496	newly	189.	2539.34	{1239., 3024.}	1243.57	0.989702	Reff	0.280894	0.412116	{0.17393, 0.750046}	74.4003	ar	88 762.	179 442.	{172 520., 194 168.}	102.161
32	R0	{1.93236, 7.38987}	{1.93126, 3.58497}	{{0.387647, 0.0177873}, {3., 11.4988}}	{65.2525, 80.5981}	inf	389.	7053.53	{3078.37, 14 814.5}	1714.41	1.04117	newly	288.	6909.94	{1426., 16 723.}	2300.01	1.18778	Reff	0.437467	0.30489	{0.0650821, 0.517944}	44.7634	ar	77 654.	194 114.	{173 751., 245 330.}	149.973
33	R0	{2.82251, 17.6954}	{2.5754, 12.5587}	{{1.63988, 6.309}, {3., 18.3705}}	{16.6785, 32.7443}	inf	4866.	1161.49	{184.437, 3321.81}	76.2723	0.833245	newly	1670.	540.59	{69., 1223.}	68.0042	0.635869	Reff	0.244147	0.563669	{0.371948, 0.798347}	133.305	ar	92 745.	155 204.	{142 972., 182 479.}	67.345
34	R0	{1.87083, 12.3994}	{1.94239, 8.99788}	{{0.480115, 2.74163}, {3., 15.7736}}	{66.1889, 47.2206}	inf	3095.	6953.76	{973.895, 16 165.1}	150.796	0.40189	newly	1528.	4465.16	{395., 7179.}	218.543	0.54685	Reff	0.498595	0.286899	{0.0329409, 0.637835}	53.921	ar	76 282.	174 967.	{164 647., 192 798.}	129.369
35	R0	{2.95779, 16.3003}	{2.4118, 9.32466}	{{1.52704, 3.34828}, {3., 17.0759}}	{20.1719, 45.8624}	inf	3818.	7017.53	{2739.25, 12 696.}	97.9649	0.205236	newly	1428.	9830.7	{1616., 33 485.}	594.472	0.50822	Reff	0.209086	0.813697	{0.306241, 1.53503}	291.408	ar	93 873.	181 303.	{159 027., 247 609.}	93.136
36	R0	{2.18718, 14.261}	{2.68285, 9.95585}	{{2.14285, 5.4298}, {3., 17.1851}}	{24.1626, 45.6888}	inf	3033.	4168.83	{1698.27, 2934.75}	56.8836	0.0972024	newly	1295.	4067.97	{719., 8017.}	236.328	0.402084	Reff	0.381269	0.887214	{0.0837734, 2.14283}	159.766	ar	84 379.	185 811.	{162 162., 244 884.}	120.209
37	R0	{2.44912, 9.31118}	{2.3929, 9.31098}	{{1.39717, 2.83568}, {3., 14.115}}	{30.1886, 52.5838}	inf	559.	3775.96	{427.312, 2178.11}	587.697	0.565431	newly	306.	3370.95	{205., 3614.}	1016.47	0.717595	Reff	0.289559	0.680689	{0.312775, 1.39779}	138.819	ar	88 442.	192 067.	{176 163., 231 192.}	117.167
38	R0	{2.05949, 5.72514}	{2.25233, 8.53886}	{{1.3769, 2.83233}, {3., 12.2214}}	{38.9598, 86.6124}	inf	81.	4475.16	{3132.64, 3132.64}	5424.88	1.61907	newly	76.	4482.15	{1489., 4889.}	5797.57	1.52356	Reff	0.380141	0.611428	{0.22011, 1.3769}	73.7147	ar	81 587.	193 335.	{180 516., 233 977.}	136.968
39	R0	{1.75505, 8.52404}	{2.39596, 8.60567}	{{1.22243, 2.2089}, {3., 15.1345}}	{57.1168, 68.6501}	inf	1029.	5069.43	{802.711, 6895.42}	408.802	0.497454	newly	719.	5328.91	{345., 7125.}	665.784	0.637153	Reff	0.527357	0.593467	{0.186545, 1.04916}	52.2091	ar	70 925.	176 301.	{161 268., 207 592.}	148.575
40	R0	{2.24724, 13.7861}	{2.53707, 5.72849}	{{1.00153, 1.52522}, {3., 8.90402}}	{29.1531, 59.4354}	inf	2420.	8936.42	{2621.44, 11 573.3}	282.588	0.585704	newly	1045.	10 749.	{2003., 24 165.}	940.95	0.907615	Reff	0.360008	0.579514	{0.076424, 1.15461}	96.3191	ar	85 257.	190 900.	{171 726., 225 799.}	123.911
41	R0	{1.77635, 8.62406}	{2.41517, 5.85278}	{{1.34714, 3.39378}, {3., 9.4633}}	{49.4557, 39.1615}	inf	882.	10 812.9	{3451.05, 41 478.4}	1128.69	0.771034	newly	608.	8396.49	{2027., 29 241.}	1283.22	0.876158	Reff	0.494927	0.470641	{0.119924, 1.03212}	50.3036	ar	72 941.	194 659.	{170 590., 288 410.}	166.872
42	R0	{1.80397, 5.4932}	{2.53587, 11.7595}	{{2.03306, 8.00687}, {3., 18.0052}}	{40.5717, 117.26}	inf	102.	7419.06	{2696.95, 10 378.1}	7173.59	1.72431	newly	82.	5907.65	{1596., 7147.}	7104.45	1.60136	Reff	0.48442	0.809668	{0.236716, 2.03304}	88.2474	ar	73 232.	202 456.	{176 064., 268 380.}	176.459
43	R0	{2.83096, 16.7077}	{1.81277, 3.67872}	{{0.0977425, 1.08319}, {3., 6.44258}}	{43.0123, 77.9819}	inf	3887.	4394.2	{114.312, 10 258.3}	102.597	0.614749	newly	1377.	4544.56	{97., 13 324.}	269.101	0.61208	Reff	0.237546	0.285693	{0.0126806, 0.727548}	99.0798	ar	92 740.	166 446.	{154 263., 199 672.}	79.4763
44	R0	{2.73678, 11.6021}	{2.68239, 10.3814}	{{2.11831, 3.94341}, {3., 12.2434}}	{14.6828, 20.7563}	inf	851.	10 105.8	{3436.65, 12 760.6}	1087.52	0.852704	newly	393.	7314.65	{1544., 22 280.}	1761.23	0.986752	Reff	0.225237	0.631137	{0.0259645, 2.11829}	221.185	ar	92 003.	207 885.	{179 080., 268 226.}	125.955
45	R0	{2.14235, 6.98601}	{2.70893, 8.06799}	{{2.02977, 2.7249}, {3., 13.5045}}	{29.5997, 62.5058}	inf	172.	9409.88	{596.922, 2943.5}	5371.07	1.26939	newly	135.	6634.	{711., 5214.}	4814.98	1.26002	Reff	0.36707	0.571966	{0.242998, 0.741729}	78.3349	ar	82 974.	196 458.	{174 359., 209 846.}	136.771
46	R0	{2.23409, 9.21289}	{2.67868, 11.494}	{{2.05493, 3.26704}, {3., 15.4867}}	{25.3532, 58.5663}	inf	866.	5836.17	{222.866, 6480.44}	602.725	0.69399	newly	520.	3569.22	{97., 7353.}	609.015	0.712175	Reff	0.341659	0.707203	{0.319298, 1.47367}	117.675	ar	85 163.	180 389.	{159 216., 208 217.}	111.816
47	R0	{2.12258, 8.15265}	{2.31725, 9.10775}	{{0.928652, 2.76089}, {3., 13.0379}}	{46.221, 46.7889}	inf	433.	7333.18	{164.995, 5406.93}	1618.83	0.91309	newly	280.	4391.42	{87., 5527.}	1497.12	0.961196	Reff	0.375293	0.483447	{0.13574, 0.923313}	59.3099	ar	82 690.	185 231.	{169 314., 208 679.}	124.006
48	R0	{2.71771, 10.4636}	{2.15016, 8.87815}	{{1.3482, 2.74351}, {3., 13.8447}}	{30.6473, 47.166}	inf	924.	5981.44	{4030.52, 4030.52}	550.889	0.705765	newly	498.	4754.84	{1680., 6415.}	858.663	0.84277	Reff	0.232147	0.554984	{0.12876, 1.35044}	157.228	ar	91 716.	196 499.	{181 361., 236 301.}	114.247
49	R0	{2.44369, 6.66477}	{2.59201, 6.50036}	{{1.8343, 4.24729}, {3., 9.01486}}	{23.5253, 33.8056}	inf	49.	2834.86	{104.066, 3763.7}	5685.94	1.5564	newly	43.	2910.83	{111., 3351.}	6669.93	1.55028	Reff	0.273693	0.45921	{0.134495, 0.96883}	104.83	ar	88 827.	172 321.	{162 527., 174 114.}	93.9963
50	R0	{2.77383, 17.9775}	{1.82523, 5.41501}	{{0.127687, 3.73638}, {3., 7.30437}}	{43.8196, 69.879}	inf	3843.	7144.24	{3552.54, 9371.61}	104.509	0.180435	newly	1236.	5811.12	{2162., 8291.}	375.827	0.514033	Reff	0.244319	0.276166	{0.0181704, 0.696333}	90.8925	ar	92 334.	170 877.	{159 209., 180 254.}	85.0641
